# Special Issue “New Frontiers in Small DNA Virus Research”

**DOI:** 10.3390/v15010259

**Published:** 2023-01-16

**Authors:** Katerina Strati, Dohun Pyeon

**Affiliations:** 1 Department of Biological Sciences, University of Cyprus, Nicosia 1678, Cyprus; 2 Department of Microbiology and Molecular Genetics, Michigan State University, East Lansing, MI 48824, USA

Scientific progress in understanding, preventing, treating, and managing viral infections and associated diseases exemplifies the extent to which research on small DNA tumor viruses has impacted human health. Various diagnostic tools for detecting virus infections and effective prophylactic vaccines against human papillomavirus (HPV) and hepatitis B virus (HBV) now promise to reduce the disease burden of these viruses in the coming decades. At the same time, these small DNA viruses, including the polyomavirus SV40 and adenoviruses, have been indispensable tools for studying molecular mechanisms of carcinogenesis and basic cellular functions. Fundamental processes of cellular biology, such as transcriptional regulation, splicing, and DNA damage, have been elucidated, at least in part, through the study of the replication cycles of such viruses and their interactions with the host. Nowadays, they also serve as valuable tools outside the research setting as viral vectors in numerous gene therapies and vaccines. Despite the advent of new technologies, research on small DNA viruses remains relevant to discovering novel biological mechanisms and clinical applications further to reduce disease burden. In this Special Issue, we have collected work that showcases current and ongoing work towards these goals.

Four studies tackle unanswered questions related to diseases caused by small DNA viruses. Sasivimolrattana et al. provide evidence that the virome in HPV-infected tissues is associated with HPV status and abundance using a metagenomic analysis [1]. As the cervical microbiota are important factors for disease progression, this study suggests further studies to ascertain the role of other viruses as cofactors in carcinogenesis and/or markers of disease progression. Jeng et al. report that deletion mutations in the HBV surface protein pre-S2, increased levels of PD-L1 expression, and regulatory T (Treg) cell infiltration into the tumor tissues may help to predict a high risk for recurrence of hepatocellular carcinoma (HCC) [2]. Next, Koubek et al. provide promising results showing that natural compounds in the schweinfurthin family can be used as chemotherapeutic agents to treat Merkel cell carcinoma (MCC) [3]. Finally, a case report by Jehn et al. shows that belatacept, an inhibitor of T cell activation through CD80/86-CD28 co-stimulatory signaling, is a potential treatment option for BK polyomavirus nephropathy (BKPyVAN) [4].

Two articles explore the basic questions in biology of two small DNA viruses: papillomavirus and adenovirus. Moreno et al. demonstrate evidence in support of the long-standing hypothesis that infected tissue progenitors (in this case, Lgr5+ cells) are preferential targets for papillomavirus-induced transformation, using the preclinical murine papillomavirus infection model [5]. Aleman et al. report the mechanism of how adenovirus shuts off host protein synthesis during adenoviral infection [6]. Their results suggest that competition at the expense of the host may be occurring at the level of mRNA biogenesis during the late stages of viral replication.

In addition to the original research articles, this Special Issue includes four review articles to update recent progress in small DNA virus research. Suresh and Menne summarize recent preclinical studies of developing antivirals, immunomodulators, and therapeutic vaccines for chronic hepatitis B, using the Eastern woodchuck with woodchuck hepatitis virus as an HBV laboratory animal model [7]. Loke et al. describe in vitro and in vivo model systems of Merkel cell polyomavirus (MCPyV) currently available for studying MCPyV-associated MCC [8]. Kines and Schiller provide an in-depth discussion about the role of heparan sulfate proteoglycans (HSPG) in HPV entry into the host cell, particularly focusing on the modification of sulfation patterns on HSPG. They suggest that HSPG can be used for the specific targeting of tumor cells using HPV virus-like particles [9]. Arman and Munger review the roles of long noncoding RNAs (lncRNAs) that dysregulate the host mechanisms to contribute to persistent HPV infection and replication in the host cell [10].

For several decades, our studies in small DNA viruses have pioneered breakthroughs in understanding fundamental molecular and cellular mechanisms. The articles published in this Special Issue demonstrate that this process continues. Yet, new questions and challenges to eradicating the diseases caused by small DNA viruses do not seem to be disappearing. While discovering detailed views of virus–host interactions and pathogenesis, we comprehend the extreme complexity of virus–host interactions and pathogenesis interweaved with other microbes and host immunity as an ecological system. In the host cell, the virus manipulates various cellular processes not fully understood, such as epigenetic regulations, noncoding RNAs and RNA modifications, posttranslational mechanisms, and metabolic pathways. In the clinic, there is an urgent need to develop novel antivirals, prophylactic and therapeutic vaccines, and biomarkers to effectively diagnose and treat patients with persistent virus infection and virus-associated diseases. Working together, we will continue towards achieving our ultimate goal of controlling virus infection and virus-associated diseases. Finally, we thank all authors for contributing to this Special Issue.

## Data Availability

No new data were created or analyzed in this study. Data sharing is not applicable to this article.
